# Supplementary material for: Substrate stiffness engineered to replicate disease conditions influence senescence and fibrotic responses in primary lung fibroblasts
Source: Front Pharmacol. 2022 Nov 3;13:989169. doi: 10.3389/fphar.2022.989169 (PMC9673045; doi:10.3389/fphar.2022.989169)
Supplement: Supplementary file 1 [file DataSheet2.docx]

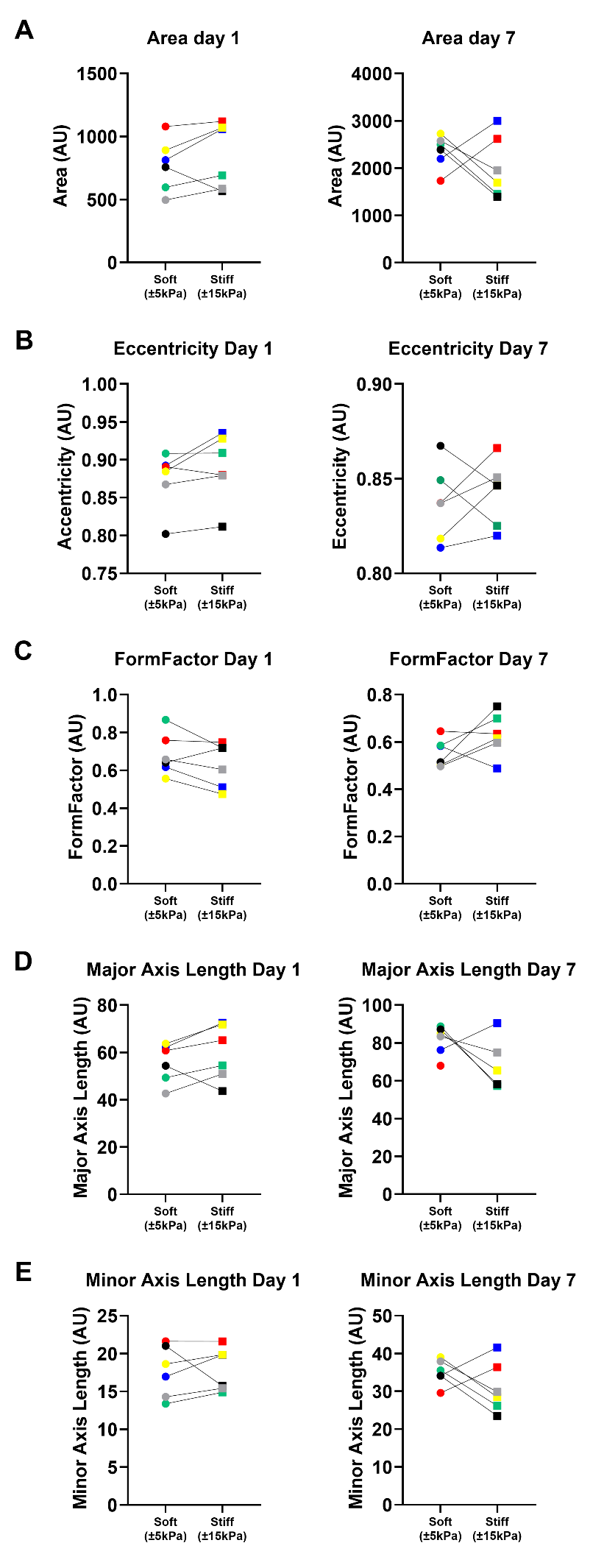

Supplementary figure 1; Cellular size and shape analysis of fibroblasts at day 1 and 7 on soft and stiff GelMA hydrogels. Quantification of cellular area (A), eccentricity (B), Formfactor (C, how defined the outline of object is) and Major and minor axis length (D-Ehow o) (n = 6). Each colour is an unique fibroblast donor (Blue, Yellow, Red, Yellow, Green, Black and Grey)
