# Supplementary material for: Substrate stiffness engineered to replicate disease conditions influence senescence and fibrotic responses in primary lung fibroblasts
Source: Front Pharmacol. 2022 Nov 3;13:989169. doi: 10.3389/fphar.2022.989169 (PMC9673045; doi:10.3389/fphar.2022.989169)
Supplement: Supplementary file 2 [file DataSheet1.docx]

| **Table 1.** Characteristics of fibroblast donors. | | | | | |
| --- | --- | --- | --- | --- | --- |
| **Donor #** | **Sex** | **Age** | **Smoking History** | **Pack Years** | **FEV1/FVC** |
| 1 | F | 49 | Current | 33 | 82.3 |
| 2 | F | 50 | Never | 0 | 77.9 |
| 3 | F | 47 | Current | 30 | 73.9 |
| 4 | F | 51 | Current | 70 | 78.3 |
| 5 | F | 49 | Ex | 35 | 77.7 |
| 6 | F | 46 | Ex | 32 | 81.5 |
| 7 | M | 69 | Current | 20 | 70.0 |
| F = female, M=male and Ex = former smoker | | | | |  |

| **Table 2.** Primers and probes with designated exon boundaries | | |
| --- | --- | --- |
| **Gene** | **Number** | **Exon boundary** |
| 18S | Hs99999901_s1 | 1-1 |
| CDKN2A | Hs00923894_m1 | 2-3 |
| CDKN1A | Hs00355782_m1 | 2-3 |
| P53 | Hs01034249_m1 | 10-11 |
| IL-6 | Hs00174131_m1 | 4-5 |
| CXCL8 | Hs00174103_m1 | 1-2 |
| DCN | Hs00370385_m1 | 7-8 |
| ACTA2 | Hs00426835_g1 | 2-3 |
| COL1A1 | Hs00164004_m1 | 1-2 |
| FN1 | Hs01549976_m1 | 8-9 |
| FBLN1 | Hs00242545_m1 | 13-14 |
| FBLN1C | Hs00242546_m1 | 14-15 |
| LOX | Hs00942483_m1 | 5-6 |
| TGF-β1 | Hs00998133_m1 | 6-7 |
| CTGF | Hs00170014_m1 | 4-5 |
| 18S ribosomal RNA = 18S, Cyclin-dependent kinase inhibitor 2A = CDKN2A, Interleukin 6 = IL-6, Chemokine ligand 8 = CXCL8, Decorin = DCN, Actin alpha 2 = ACTA2, Collagen 1α1 = COL1A1, Fibronectin = FN1, Fibulin-1 = FBLN1, Lysyl oxidase = LOX, Transforming growth factor β1 = TGF-β1, Connective tissue growth factor = CTGF | | |
